# Social support mediates the relationship between illness perception and psychosocial adaptation among young and middle-aged kidney transplant recipients in China

**DOI:** 10.3389/fpsyg.2023.1062337

**Published:** 2023-02-24

**Authors:** Na Hu, Aiping Wang, Tiantian Chang

**Affiliations:** ^1^Transplantation Department, The First Affiliated Hospital of China Medical University, Shenyang, Liaoning, China; ^2^Department of Public Service, The First Affiliated Hospital of China Medical University, Shenyang, Liaoning, China

**Keywords:** young and middle-aged, kidney transplant recipients, psychosocial adaptation, illness perception, social support, mediating effect

## Abstract

**Background:**

No research has yet been done on social support’s influence on the association between illness perception and psychosocial adaptation among young and middle-aged kidney transplant recipients in China. Accordingly, it remains unclear how medical personnel can assist patients in successfully adjusting to the early postoperative period and improving their health.

**Objective:**

This study sought to explore the influence of illness perception and social support on the psychosocial adaptation of young and middle-aged recipients of kidney transplants in China during the early postoperative period.

**Methods:**

This study adopted a cross-sectional design. The study included 236 young and middle-aged kidney transplant recipients from a tertiary hospital in China. Demographic and disease-related data were collected. Additionally, the Psychosocial Adjustment to Illness Scale-Self-Report, the Brief Illness Perception Questionnaire, and the Multidimensional Scale of Perceived Social Support were used to assess participants’ psychosocial adaptation, illness perception, and social support, respectively. The model was examined using descriptive analysis, Pearson’s correlation analysis, hierarchical multiple regression analysis, and the PROCESS Macro in SPSS 26.0.

**Results:**

A total of 176 (74.56%) participants reported an average psychosocial adaptation score >50, which is relatively negative. Marital status, education level, residence, *per capita* monthly income (in Chinese yuan), medical insurance, work status, post-transplant time, body mass index, creatinine status, and complications were all related to psychosocial adaptation (*p* < 0.05). The more negative their illness perception and the worse their social support, the worse the psychosocial adaptation of young and middle-aged kidney transplant recipients. Further, the effect of illness perception on psychosocial adaptation was partially mediated by social support (36.56%).

**Conclusion:**

In general, the psychosocial adaption level of young and middle-aged kidney transplant recipients was negative during the early postoperative period. Healthcare teams should assist patients in building a positive illness perception shortly following kidney transplantation, while also providing psychological care and support to help them cope with the onset of psychosocial issues.

## 1. Introduction

Chronic kidney disease (CKD) is a major global public health concern, and its prevalence ranges from 11.7 to 15.1% worldwide (Lv and Zhang, 2019). Some researchers estimate that over 434 million Asians suffer from CKD, while China exhibits the highest prevalence of the disease, with approximately 159.8 million individuals afflicted (Liyanage et al., 2022). When a patient’s kidney function gradually declines, end-stage renal disease (ESRD) develops. Currently, the most common ESRD treatment options are kidney transplantation, hemodialysis, and peritoneal dialysis. Kidney transplantation is the optimal treatment for patients with ESRD who would otherwise require dialysis (Stoumpos et al., 2015). However, aging is frequently associated with multiple comorbidities and lower physical status, making kidney transplantation candidates less surgically fit (Pravisani et al., 2021). Determining who among older kidney transplant candidates is appropriate for transplantation can be challenging and complex (Concepcion et al., 2016). According to Jankowska et al. (2021), the long-term patient and graft survivals were poorer in the elderly kidney transplant recipients group versus the younger kidney transplant recipients group from the same donor. Additionally, 75% of deceased donor transplant recipients aged 30–49 years are alive after 5 years compared to only 61% for those older than 65 years (Knoll, 2013). Previous research has revealed that the younger are more eligible for transplant than the elderly who have co-morbidities with poor prognosis and more cardiovascular complications predominantly (Singh et al., 2016; Lim et al., 2022). A thorough medical evaluation with particular focus on cardiovascular health must be employed (Concepcion et al., 2016). Therefore, this study chooses the young and middle-aged population as the research object. Although kidney transplant surgery has many benefits, such as improving quality of life and facilitating metabolic function recovery at a lower cost than other renal replacement therapies (Thuret et al., 2016), patients who are unable to manage the postoperative challenges of kidney transplantation face a variety of psychosocial health issues.

The concept of adaptation was originally proposed in the field of biology (Darwin and Field, 2011) but has since been extended to the fields of psychology and nursing, as well as to society at large. Notably, scholars such as Londono and McMillan (2015) used Rodger’s evolutionary perspective to analyze the concept of psychosocial adaptation. Delineating psychosocial adaptation requires an understanding of psychological and social adaptation. According to previous research, “psychological adaptation” is a self-regulating process characterized by the use of psychological defense mechanisms to reduce stress and maintain balance after experiencing setbacks; meanwhile, “social adaptation” involves altering behavior to conform to social expectations in a way that balance the individual’s relationship with their environment (Derogatis, 1986; Livneh and Antonak, 2005; Yao, 2013; Yuan, 2018; Livneh, 2022). Generally, psychosocial adaptation to illness refers to the patient’s emotional experience, self-evaluation, and attitude after illness, as well as the process of adjusting behaviors to various social groups and norms (Yao, 2013; Londono and McMillan, 2015; Xu et al., 2022). The psychosocial adaption of kidney transplant recipients is a dynamic process impacted by a variety of factors and is closely related to quality of life (Pasquale, 2014; Kamran and Ogden, 2016). According to Wurm et al. (2022), kidney transplant recipients have adverse psychosocial experiences after surgery and often have more unmet needs related to equality in healthcare, acceptance, and negative emotion. Indeed, prior research found that the prevalence of anxiety ranged from 25 to 50% among kidney transplant recipients (Fanakidou et al., 2017). Research also indicates that 42.4% of kidney transplant recipients experience moderate-to-severe psychological issues (Tian et al., 2016). Additionally, long-term emotional problems increase the risk of mortality, graft rejection, and readmission (Molnar-Varga et al., 2011; Zelle et al., 2012). Researchers analyzed 29,809 recipients of kidney transplants using the United Network for Organ Sharing database and found that only 47% returned to work 1 year after surgery; such a delay in the resumption of normal activities is bound to have an effect on physical and mental health (Tzvetanov et al., 2014). Further, in China, 61.6% of female kidney transplant recipients experience sexual dysfunction and sexual activity avoidance; a decrease in the level of sexual desire and general dissatisfaction with sexual life can lead to impaired social function and various mental health problems (Gonçalves et al., 2019; Xiao et al., 2021). Therefore, the psychosocial adaptation of young and middle-aged kidney transplant recipients should be explored. In response, this study examined the postoperative psychosocial adaptation of such individuals in China.

“Illness perception” refers to a patient’s cognitive evaluation of their disease, as well as their understanding of the medical condition and its potential consequences throughout the disease course (Broadbent et al., 2015). Positive illness perception is conducive to favorable health outcomes, whereas negative illness perception can impair the individual’s ability to cope with disease and, Consequently, influence their perception of the disease as manageable or dangerous (Bonsaksen et al., 2015; Sawyer et al., 2019). Previous research has revealed that kidney transplant recipients who perceive their condition more negatively report lower quality of life and more maladaptive coping strategies (Knowles et al., 2016; Kalfoss et al., 2019). Additionally, other researchers noted that medication non-compliance was reported by 32.4% of kidney transplant recipients, while having a positive outlook on one’s health improved medication adherence and patient outcomes (Massey et al., 2013; Wang et al., 2022). According to Zelikovsky and Nelson (2021), an adolescent kidney transplant recipient’s negative beliefs and perceptions about their illness negatively impact their well-being beyond the physical effects of their illness and medications. Thus, negative illness perception has a significant impact on patients’ postoperative health outcomes. However, no studies have been done on illness perception and psychosocial adaptation in kidney transplant recipients. Consequently, this study explored young and middle-aged kidney transplant recipients’ illness perceptions at the early postoperative stage to determine whether illness perception influences their psychosocial adaptation.

“Social support” is a multidimensional term that pertains to the availability of social resources in a given circumstance (Cipolletta et al., 2019); notably, it can orginate from a range of sources, including the individual’s family, friends, and colleagues (Zimet et al., 1990). When kidney transplant recipients receive a high degree of family care and social support (from friends, family, etc.), social support can have a buffering effect by regulating stress and anxiety and can thus improve postoperative mental health (Pisanti et al., 2014; Mouelhi et al., 2018). Previous research has shown that good social support can have a positive impact on patients’ quality of life following surgery; indeed, the same holds true for positive illness perception (Li et al., 2016; Sitjar-Suñer et al., 2020). Along these lines, scholars have found that insufficient levels of social support and negative illness perception can worsen the disease prognosis of patients with ESRD (Suarilah and Lin, 2021; Gunarathne et al., 2022). However, the association between illness perception, social support, and psychosocial adaptation among young and middle-aged kidney transplant recipients in the early postoperative period is currently unknown. Consequently, this study hypothesized that social support plays a mediating role between illness perception and psychosocial adaptation. Specifically, we proposed four hypotheses: (1) positive illness perception is positively associated with positive psychosocial adaptation, (2) negative illness perception negatively affects social support, (3) positive social support is positively associated with positive psychosocial adaptation, and (4) negative illness perception negatively and indirectly influences psychosocial adaptation *via* social support.

## 2. Materials and methods

### 2.1. Design and sampling

This study used a cross-sectional design. Two hundred and thirty-six patients who met the inclusion criteria were recruited from the transplantation department of the First Affiliated Hospital of China Medical University between October 2020 and September 2022. The inclusion criteria comprised the following: (1) patients between 18 and 59 years of age, (2) patients who underwent their first kidney transplant less than a year prior, and (3) patients who are cognizant, able to read and communicate, and who have provided their informed consent to actively engage in this study. Exclusion criteria comprised the following: (1) patients who underwent two or more organ transplants concurrently and (2) patients who had severe psychiatric or cognitive diseases.

### 2.2. Questionnaires

#### 2.2.1. Demographic and disease characteristics

The general information section of the questionnaire mainly included two parts: demographic data and disease-related data. Demographic data included gender, age, marital status, education level, residence, *per capita* monthly income (in Chinese yuan [CNY]), medical insurance status, and employment status. Disease-related data included post-transplant time, type of preoperative dialysis, kidney source, body mass index (BMI), creatinine level, and post-transplant complications.

#### 2.2.2. The psychosocial adjustment to illness scale—self-report

The PAIS-SR was designed by Derogatis (1986) to evaluate patients’ psychosocial adaptation. Yao (2013) modified the original scale by deleting two items—their final Chinese version comprises 44 items across seven dimensions (work ability, health care, family relations, sexual ability, communication, entertainment, and psychological status); each item is scored on a 4-point scale ranging from 0 to 3, with the total score ranging from 0 to 132 points. Specifically, 34 points indicate good psychosocial adaptation, 34 to 50 points indicate medium psychosocial adaptation, and > 50 points indicate poor psychosocial adaptation, with a higher score indicating worse psy-chosocial adaptation. The PAIS-SR has proven to have excellent reliability and validity in Chinese samples (Yuan, 2018). In the current study, its Cronbach’s alpha was 0.915.

#### 2.2.3. The brief illness perception questionnaire

The BIPQ was designed by Broadbent et al. (2006) to rapidly evaluate patients’ perceptions of their illnesses. The scale comprises eight items. Each item is scored on a scale of 0 to 10, for a total of 80 points (items 3, 4, and 7 are reverse-scored). The higher the score, the more severe the patient’s negative perception of the disease, and the greater the perceived illness threat; 16–32 indicates mild illness perception, 32–48 indicates moderate illness perception, 48–64 indicates moderate-to-severe illness perception, and > 64 indicates severe illness perception. The reliability and validity of the scale are acceptable among Chinese samples (Massey et al., 2013; Zelikovsky and Nelson, 2021). In the current study, the scale’s Cronbach’s alpha was 0.890.

#### 2.2.4. The multidimensional scale of perceived social support

The MSPSS was designed by Zimet et al. (1990) to measure perceived individual social support. It includes three dimensions: family, friends, and support from others. Each of the 12 items is scored on a scale ranging from 1 (“totally disagree”) to 7 (“totally agree”). The total score ranges from 12 to 84, and it indicates the level of social support; the higher the score, the higher the level of social support. The scale has been demonstrated to have good validity and reliability in Chinese samples (Du et al., 2018). In the current study, the Cronbach’s alpha for the MSPSS scale was 0.866. The Cronbach coefficients for the scale’s family, friends, and other supporting dimensions were 0.861, 0.882, and 0.830, respectively.

### 2.3. Data collection procedure

The data was collected face-to-face. Before distributing the questionnaire, the researcher explained the purpose, significance, and procedure of the study to the participants, who then signed an informed consent form after being guaranteed that their privacy would be safeguarded. This study was conducted in accordance with the Declaration of Helsinki and involved no animal testing. All procedures were approved by the ethics committee of the research center. The questionnaires were filled out anonymously, and all participants were assured that their participation was fully voluntary, that they could withdraw at any time, and that their data would be deleted if they withdrew from the study. The researchers assured the participants that their identities and responses would be kept confidential and that a truthful answer would not impact their work. In total, 248 questionnaires were sent out, of which 236 were returned (recovery rate of 95.2%).

### 2.4. Statistical analysis

Statistical analyses were performed using SPSS software, version 26.0. All tests were two-tailed, with *p* < 0.05 as the threshold for statistical significance. The distribution of psychological adaptation across demographic and disease factors was examined using nonparametric testing. Ten variables were selected as control variables in the multivariate hierarchical model based on the univariate analysis (*p* < 0.05) and clinical significance (Wang et al., 2021). The relationship between illness perception, social support, and psychosocial adaptation was determined using Pearson correlations. A hierarchical multiple regression analysis examined social support as a potential mediator between illness perception and psychosocial adaptation. Model 4 in the PROCESS was selected to test the mediation effect. First, control variables were input (Block 1). Second, illness perception was added (Block 2). Finally, social support was added as a mediator (Block 3). To further assess the mediating role of social support, 5,000 bootstrap samples were used *via* the PROCESS macro. If the bias-corrected and accelerated 95% confidence interval (CI) did not contain 0, there was a significant mediating effect.

## 3. Results

### 3.1. Participant characteristics

Among the 236 patients, the mean age was 39.97 ± 9.26 (20–59 years). Table 1 shows the demographic and clinical characteristics of the sample population and the results of the univariate model, including marital status, education level, residence, *per capita* monthly income (in Chinese yuan), medical insurance, work status, post-transplant time, BMI, creatinine status, and complications. All these characteristics were related to psychosocial adjustment (*p* < 0.05); therefore, they were entered as control variables in the hierarchical multiple linear regression model.

**Table 1:** Demographic and clinical characteristics by psychosocial adaptation and results of the univariate model.

Variables	Psychosocial adaptation		*Z*	*p*
	*N*	*M* ± SD		
Gender			0.084	0.933
Male	144 (61.02)	65.25 ± 16.13		
Female	92 (38.98)	65.07 ± 16.88		
Age			1.508	0.133
18–< 40	122 (51.69)	66.73 ± 16.50		
40–60	114 (48.31)	63.52 ± 16.18		
Marital status			3.053	0.003
Single/Divorced/Widowed	109 (46.19)	68.63 ± 16.42		
Married	127 (53.81)	62.21 ± 15.84		
Education level			6.141	0.003
High school or below	80 (33.90)	69.60 ± 18.13		
Junior college	117 (49.58)	64.21 ± 14.98		
Bachelor degree or above	39 (16.52)	59.00 ± 14.48		
Residence			−3.126	0.002
Urban	152 (64.41)	62.74 ± 15.91		
Rural	84 (35.59)	69.58 ± 16.43		
*Per capita* monthly income (in Chinese yuan)			4.737	0.010
< 6,000	119 (50.42)	67.91 ± 16.08		
6,000-10,000	87 (36.86)	63.82 ± 16.08		
> 10,000	30 (12.72)	58.30 ± 16.55		
Medical insurance			3.928	0.021
No insurance	70 (29.66)	69.67 ± 15.79		
Urban resident basic medical insurance	118 (50.00)	62.93 ± 15.51		
New rural cooperative medical system	48 (20.34)	64.15 ± 18.30		
Work status			11.079	0.000
Returned to work	66 (27.97)	58.42 ± 15.58		
Have a job but not returned to work	92 (38.98)	65.25 ± 13.91		
Unemployed	78 (33.05)	70.81 ± 17.76		
Post-transplant time			15.151	0.000
< 3 months	49 (20.76)	73.35 ± 18.05		
3–6 months	50 (21.19)	69.82 ± 16.38		
6–12 months	137 (58.05)	60.56 ± 14.11		
Types of preoperative dialysis			1.456	0.235
Peritoneal dialysis	70 (29.66)	63.93 ± 16.34		
Hemodialysis	128 (54.24)	64.65 ± 14.87		
Undialyzed	38 (16.10)	69.26 ± 20.70		
Kidney source			−1.197	0.233
Hospital donation	207 (87.71)	64.70 ± 16.57		
Living relative donor	29 (12.29)	68.59 ± 14.85		
BMI			2.967	0.003
Abnormal	86 (36.44)	69.29 ± 18.03		
Normal	150 (63.56)	62.82 ± 14.93		
Creatinine status			−5.212	0.000
Stable	135 (57.20)	60.61 ± 14.19		
Unstable	101 (42.80)	71.28 ± 17.20		
Complication			4.084	0.000
Yes	192 (81.36)	67.20 ± 16.02		
No	44 (18.64)	56.36 ± 15.19		

BMI = body mass index; *M* = mean; SD = standard deviation.

### 3.2. Correlations between illness perception, social support, and psychosocial adaptation

Table 2 shows that illness perception and social support were correlated with psychosocial adaptation. Social support was negatively correlated with illness perception (*r* = −0.36, *p* < 0.001) and psychosocial adaptation (*r* = −0.68, *p* < 0.001). Furthermore, illness perception was positively associated with psychosocial adaptation (*r* = 0.43, *p* < 0.001).

**Table 2:** Means, SDs, and correlations of all variables.

Variables	*M*	SD	Illness perception	Social support
Illness perception	38.89	8.84	1	
Social support	42.37	7.47	−0.36^***^	1
Psychosocial adaptation	65.18	16.39	0.43^***^	−0.68^***^

^***^p < 0.001 (two-tailed).

### 3.3. The result of the hierarchical multiple regression

As shown in Table 3, illness perception and social support each explained 16% of the variance in psychosocial adaptation. Illness perception was positively correlated with psychosocial adjustment (*β* = 0.42, *p* < 0.001) and social support was negatively correlated with psychosocial adjustment (*β* = −0.48, *p* < 0.001). When social support was added in Step 3, the regression coefficient of illness perception on psychosocial adjustment decreased from 0.42 to 0.27, suggesting that social support may act as a mediator between illness perception and psychosocial adjustment in patients. The results of this statistical analysis are consistent with this study’s hypotheses.

**Table 3:** Hierarchical multiple linear regression analysis results.

Variables	Psychosocial adaptation		
	Step 1 (*β*)	Step 2 (*β*)	Step 3 (*β*)
**Block 1**			
Marital status	−0.10	−0.06	−0.01
Education level	−0.14^*^	−0.18^**^	−0.10^*^
Residence	0.06	0.03	0.05
*Per capita* monthly income (CNY)	−0.07	−0.09	−0.04
Medical insurance	−0.09	−0.15^**^	−0.15^**^
Work status	0.06	0.04	−0.04
Post-transplant time	−0.23^***^	−0.20^***^	−0.15^**^
BMI	−0.02	−0.04	−0.03
Creatinine status	0.21^**^	0.14^*^	0.08
Complication	−0.13^*^	−0.18^**^	−0.15^**^
**Block 2**			
Illness perception		0.42^***^	0.27^***^
**Block 3**			
Social support			−0.48^***^
*R* ^2^	0.29	0.45	0.61
Δ*R*^2^	0.29	0.16	0.16

^*^p < 0.05; ^**^p < 0.01 (two-tailed) ^***^p < 0.001.

### 3.4. Results of the path analysis

**Figure 1:**
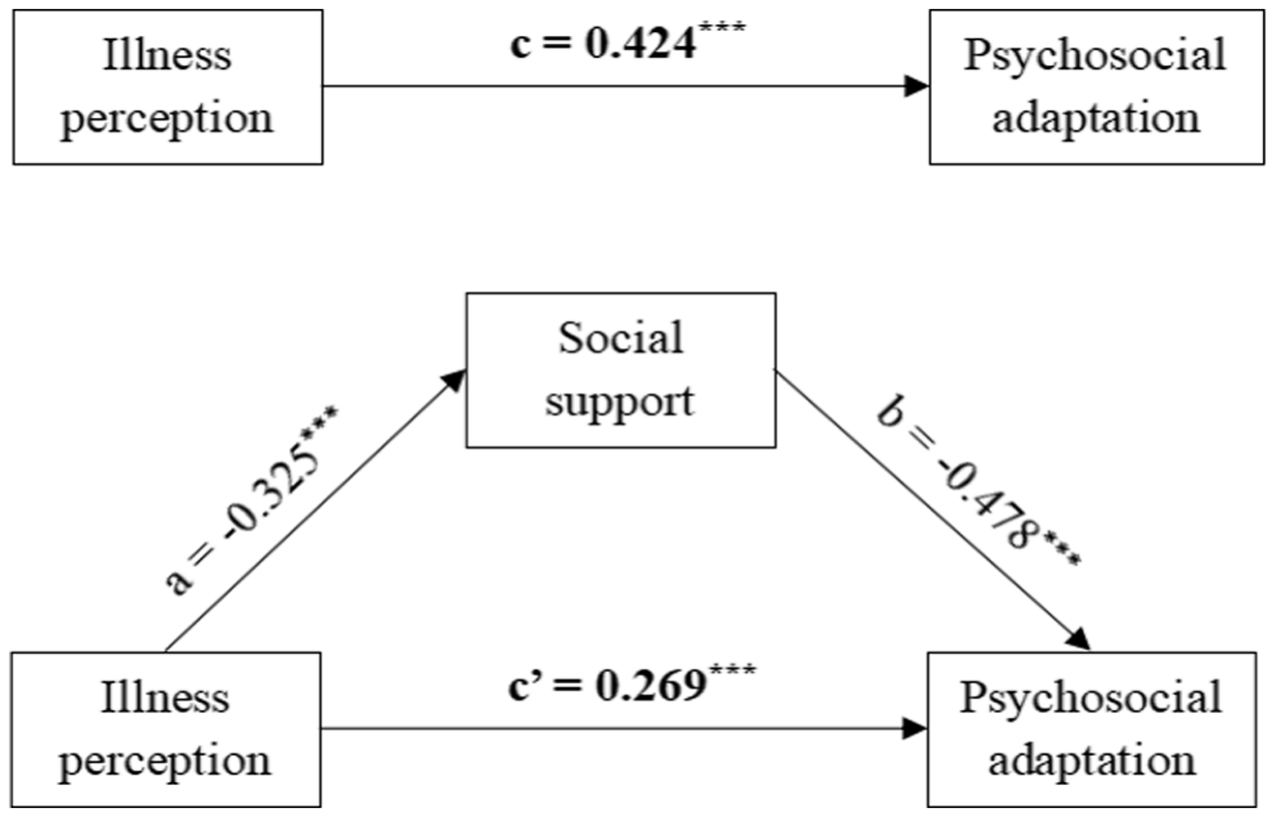
The hypothesized mediation model relating the effect of illness perception on physcosocial adoptation through social support. ***p <0.001 (two-tailed).

As shown in Table 4, using psychosocial adaptation as the dependent variable, illness perception as the independent variable, and social support as the mediating variable, an analysis of mediating effects was conducted using the nonparametric percentile bootstrap method with a set sampling of 5,000 times to test the 95% CI of each path coefficient. The path coefficients are shown in Figure 1. First, the total effect of illness perception on psychosocial adaptation (Path c) was 0.424 (*p* < 0.001). The coefficients of Paths a, b, and c were-0.325 (*p* < 0.001), −0.478 (*p* < 0.001), and 0.269 (*p* < 0.001), respectively, which suggests that illness perception, social support, and psychosocial adaptation were independently related. Furthermore, the indirect effect of illness perception on psychosocial adaptation through social support was 0.155 (*p* < 0.001, 95%CI: 0.065–0.280), which suggests that the relationship between illness perception and psychosocial adaptation (a × b) was partly mediated by social support. Finally, to understand the effect size of the mediating pathway, we used the formula (a × b)/c to calculate the proportion of the indirect effect of social support, accounting for the total effect of illness perception on psychosocial adaptation, resulting in a mediating effect of 36.56%.

**Table 4:** Mediating effect analysis.

Model	*β*	SE	*p*-value	Bootstrap 95% CI		Mediation (%)
				CI.L	CI.U	
Total effect	0.424	0.052	< 0.001***	0.322	0.527	
Direct effect	0.269	0.047	< 0.001***	0.177	0.361	63.44%
Indirect effect	0.155	0.054	< 0.001***	0.065	0.280	36.56%

^***^p < 0.001 (two-tailed).

## 4. Discussion

To the best of our knowledge, this is the first study to investigate the relationship between illness perception, social support, and psychosocial adaptation in young and middle-aged kidney transplant recipients during the early postoperative period. It clarifies that social support serves as a partial mediator between illness perception and psychosocial adaptation.

Most patients in this study received their kidneys from hospital donations. The postoperative creatinine levels of young and middle-aged kidney transplant recipients were generally stable. In addition, 81.36% of young and middle-aged kidney transplant recipients experienced complications within 1 year post-surgery, which is consistent with previous research, while the readmission rate of kidney transplant recipients within 1 year was 43.2%; the most common complications are infections, diarrhea, and diabetes (Galindo Sacristán et al., 2013; Sharma et al., 2021). As healthcare workers, we can teach patients how to recognize the symptoms of postoperative complications and encourage them to seek medical help as soon as possible.

Furthermore, the psychosocial adaptation score of young and middle-aged kidney transplant recipients after early surgery was (65.18 ± 16.39) (> 50 points), which indicates a negative psychosocial adaptation level. The study population exhibited more negative psychosocial adaptation scores, compared with hemodialysis patients (61.32 ± 16.32), possibly due to individual differences (Vicdan and Karabacak, 2016). Consequently, it is critical for medical staff to use assessment tools to determine the psychosocial adaptation level of young and middle-aged kidney transplant recipients as soon as possible.

Participants’ illness perception score was (38.89 ± 8.84), which indicates a moderately negative level. Our study is the first to reveal that the more positive the illness perception of young and middle-aged kidney transplantation recipients, the better their psychosocial adaptation level. The results of this study are consistent with those of previous studies. For example, according to Marcos et al. (2007), illness identity is associated with emotional and psychosocial adaptation, while having faith in the possibility that treatment will control the illness is associated with positive outcomes. According to Xu et al. (2022), positive illness perception, favorable disease management ability, and family function are conducive to enhancing the psychosocial adaptation level of patients with inflammatory bowel disease. Similarly, patients with hepatitis C who have a positive illness perception are more confident in their abilities to deal with disease-related challenges and have better physical and mental health outcomes (Langston et al., 2018). Conversely, other researchers believe that psychoeducational interventions can reduce the negative illness perception of kidney transplant recipients, hence enhancing medication adherence and quality of life (Látos et al., 2022; Wang et al., 2022). These findings imply that healthcare teams should assist kidney transplant recipients in identifying the causes of their negative illness perceptions and then utilize psychological intervention techniques to help them develop a more positive attitude about their condition; for example, they encourage patients to be optimistic about the future and to feel a sense of control over their lives following transplantation.

This study also revealed that the greater the level of social support for young and middle-aged kidney transplant recipients, the greater their psychosocial adaptation. Patients undergoing kidney transplants face a high risk of developing mental health issues that can negatively impact their quality of life and increase their likelihood of rejection (Chisholm-Burns et al., 2009; Pasquale et al., 2020). Researchers have noted the connection between enhanced family support and patients’ increased self-efficacy and psychosocial adaptation (Choi et al., 2012; Zhang et al., 2019). In order to help kidney transplant recipients improve their self-management skills, prolong graft function, and maintain psychological well-being, clinical nurses should concentrate on meeting their emotional and social support needs (Pisanti et al., 2014; Been-Dahmen et al., 2018). Some researchers have pointed out that through the adoption of a kidney transplant patient support program, sharing disease experiences may improve patients’ postoperative loneliness and assists them in increasing their self-confidence after kidney transplantation (Pomey et al., 2021). Medical staff should provide appropriate medical support services, while clinical nurses should fully mobilize the patient’s family members to encourage them to actively participate in the recovery and care of the kidney transplant recipient.

Moreover, illness perception not only has a direct effect on psychosocial adaptation, but it also has an indirect effect through social support. The Kidney Disease Improving Global Outcomes recommendations specify that all kidney transplant recipients must have induction therapy to prevent rejection, unless the recipient and donor are identical twins (Chadban et al., 2020). Lifetime medicine, frequent follow-ups, and considerable economic burdens affect the physical and psychological health of kidney transplant recipients (Pisanti et al., 2014; Megawati et al., 2019). According to Segal et al. (2021), there is a correlation between illness perception and social support; when illness perception is more negative, the patient’s self-confidence is lower, which makes it more difficult to control the disease, whereas the lack of social support will make it difficult to establish a correct illness perception, resulting in serious psychosocial problems. Accordingly, Grewal et al. (2010) called for a focus on early detection and interventions to enhance patients’ illness perception to simultaneously achieve risk behavior reduction and secondary prevention. The reason for this relationship could be that when young and middle-aged kidney transplant recipients develop a positive and correct illness perception, they can help improve psychosocial adaptability and promote health by strengthening the level of social support (such as from family, friends, medical care, etc.). These are all possible explanations for the correlation between illness perception, social support, and psychosocial adaptation. The results of this study are relevant for medical staff and public health departments seeking to combine the disciplines of medicine and psychology to promote multidisciplinary cooperation while providing an excellent social support system with new perspectives for enhancing postoperative psychosocial adaptation.

## 5. Conclusion

In general, the psychosocial adaptation of young and middle-aged kidney transplantation recipients is affected by many factors. The current study employed PAIS-SR, BIPQ, and MSPSS to assess psychological adaptation, illness perception, and social support of 236 kidney-transplant recipients. The statistical analyses performed found a statistically significant correlation between all three variables. A path analysis also revealed that the relationship between illness perception and psychosocial adaptation was partly mediated by social support. These results imply that clinical nurses and psychologists should focus on the psychosocial adaptation of young and middle-aged recipients following early kidney transplantation, particularly those in the early postoperative period(<1 year post-surgery). To prolong graft function, the medical team should educate patients to cooperate in timely check-ups and live a healthy lifestyle. Furthermore, kidney transplant recipients should be guided to develop positive illness perception and identify the sources of negative emotions in the early postoperative period. Simultaneously, multidisciplinary collaborative interventions should be strengthened to assist early kidney transplant recipients in successfully adapting to post-transplant changes, rebuilding social roles, return to work or school as early as possible, and improving their psychosocial adaptation.

## 6. Strengths and limitations

To our konwledge, this study is the first to explore the mediating effect of social support on the association between psychological adaptation, and illness perception among young and middle-aged kidney transplantation recipients. Through quantitative analysis, it is expected that medical staff can effectively alter the status of young and middle-aged kidney transplantation recipients’ psychological adaptation through the intervention of mediating variables, to promote their physical and mental health. Nevertheless, this study had some limitations. First, its setting, sample size, and scope were limited in that it was conducted in only one hospital. Second, this study only included young and middle-aged kidney transplant recipients; elderly kidney transplant recipients should be included in future studies. Third, this study was a cross-sectional study. While this approach allows us to determine correlations between variables, it does not offer insights into their causal relationships. A longitudinal study can be added to a future investigation to illustrate the causal relationship more clearly between variables. In addition, longitudinal studies can compare and analyze the variability and trends of psychosocial adaptation at different points in time. Fourth, we only examined the effect of social support on illness perception and psychosocial adaptation. Therefore, it is necessary to include more diverse mediating or moderating factors in future studies.

## Data availability statement

The datasets presented in this study can be found in online repositories. The names of the repository/repositories and accession number(s) can be found in the article/supplementary material.

## Ethics statement

The research proposal was approved by the Ethics Committee of the First Affiliated Hospital of China Medical University. Participants provided written informed consent.

## Author contributions

NH and AW designed the study, analyzed the data, and revised the manuscript. NH and TC collected the data. NH wrote the manuscript. All authors contributed to the article and approved the submitted version.

## Conflict of interest

The authors declare that the research was conducted in the absence of any commercial or financial relationships that could be construed as a potential conflict of interest.

## Publisher’s note

All claims expressed in this article are solely those of the authors and do not necessarily represent those of their affiliated organizations, or those of the publisher, the editors and the reviewers. Any product that may be evaluated in this article, or claim that may be made by its manufacturer, is not guaranteed or endorsed by the publisher.

## References

[ref1] Been-Dahmen J. M. J. Grijpma J. W. Ista E. Dwarswaard J. Maasdam L. Weimar W.. (2018). Self-management challenges and support needs among kidney transplant recipients: a qualitative study. J. Adv. Nurs. 74, 2393–2405. doi: 10.1111/jan.13730, PMID: 29869342

[ref2] Bonsaksen T. Lerdal A. Fagermoen M. S. (2015). Trajectories of illness perceptions in persons with chronic illness: an explorative longitudinal study. J. Health Psychol. 20, 942–953. doi: 10.1177/1359105313504235, PMID: 24140616

[ref3] Broadbent E. Petrie K. J. Main J. Weinman J. (2006). The brief illness perception questionnaire. J. Psychosom. Res. 60, 631–637. doi: 10.1016/j.jpsychores.2005.10.020 16731240

[ref4] Broadbent E. Wilkes C. Koschwanez H. Weinman J. Norton S. Petrie K. J. (2015). A systematic review and meta-analysis of the brief illness perception questionnaire. Psychol. Health 30, 1361–1385. doi: 10.1080/08870446.2015.1070851, PMID: 26181764

[ref5] Chadban S. J. Ahn C. Axelrod D. A. Foster B. J. Kasiske B. L. Kher V.. (2020). KDIGO clinical practice guideline on the evaluation and Management of Candidates for kidney transplantation. Transplantation 104, S11–S103. doi: 10.1097/tp.0000000000003136, PMID: 32301874

[ref6] Chisholm-Burns M. A. Spivey C. A. Wilks S. E. (2009). Social support and immunosuppressant therapy adherence among adult renal transplant recipients. Clin. Transpl. 24, 312–320. doi: 10.1111/j.1399-0012.2009.01060.x, PMID: 19694770

[ref7] Choi W. H. H. Lee G. L. Chan C. H. Y. Cheung R. Y. H. Lee I. L. Y. Chan C. L. W. (2012). The relationships of social support, uncertainty, self-efficacy, and commitment to prenatal psychosocial adaptation. J. Adv. Nurs. 68, 2633–2645. doi: 10.1111/j.1365-2648.2012.05962.x, PMID: 22360348

[ref8] Cipolletta S. Entilli L. Nucci M. Feltrin A. Germani G. Cillo U.. (2019). Psychosocial support in liver transplantation: a dyadic study with patients and their family caregivers. Front. Psychol. 10:2304. doi: 10.3389/fpsyg.2019.02304, PMID: 31649602 PMC6795706

[ref9] Concepcion B. P. Forbes R. C. Schaefer H. M. (2016). Older candidates for kidney transplantation: who to refer and what to expect? World J. Transplant. 6, 650–657. doi: 10.5500/wjt.v6.i4.650, PMID: 28058214 PMC5175222

[ref10] Darwin C. Field R. (2011). The Origin of Species by Means of Natural Selection: Or, the Preservation of Favored Races in the Struggle for Life. Gadsden, AL: Blackstone Pub., PMID: 26148125

[ref11] Derogatis L. R. (1986). The psychosocial adjustment to illness scale (PAIS). J. Psychosom. Res. 30, 77–91. doi: 10.1016/0022-3999(86)90069-3 3701670

[ref12] Du C. Wu S. Liu H. Hu Y. Li J. (2018). Correlation of long-term medication behaviour self-efficacy with social support and medication knowledge of kidney transplant recipients. Int. J. Nurs. Sci. 5, 352–356. doi: 10.1016/j.ijnss.2018.09.009, PMID: 31406847 PMC6626269

[ref13] Fanakidou I. Zyga S. Alikari V. Tsironi M. Stathoulis J. Theofilou P. (2017). Mental health, loneliness, and illness perception outcomes in quality of life among young breast cancer patients after mastectomy: the role of breast reconstruction. Qual. Life Res. 27, 539–543. doi: 10.1007/s11136-017-1735-x, PMID: 29119452

[ref14] Galindo Sacristán P. Pérez Marfil A. Osorio Moratalla J. M. de Gracia Guindo C. Ruiz Fuentes C. Castilla Barbosa Y. A.. (2013). Predictive factors of infection in the first year after kidney transplantation. Transplant. Proc. 45, 3620–3623. doi: 10.1016/j.transproceed.2013.11.009 24314976

[ref15] Gonçalves P. Loureiro L. Fernandes M. (2019). Sexual function of kidney transplant recipients. Rev. Enf. Ref. IV Série, 47–58. doi: 10.12707/riv19009

[ref16] Grewal K. Stewart D. E. Grace S. L. (2010). Differences in social support and illness perceptions among south Asian and Caucasian patients with coronary artery disease. Heart Lung 39, 180–187. doi: 10.1016/j.hrtlng.2009.06.016, PMID: 20457337

[ref17] Gunarathne T. G. N. S. Tang L. Y. Lim S. K. Nanayakkara N. Damayanthi H. D. W. T. Abdullah K. L. (2022). Factors associated with symptom burden in adults with chronic kidney disease undergoing hemodialysis: a prospective study. Int. J. Environ. Res. Public Health 19:5540. doi: 10.3390/ijerph19095540, PMID: 35564935 PMC9105408

[ref18] Jankowska M. Bzoma B. Małyszko J. Małyszko J. Słupski M. Kobus G. (2021). Early outcomes and long-term survival after kidney transplantation in elderly versus younger recipients from the same donor in a matched-pairs analysis. Medicine 100:e28159. doi: 10.1097/MD.0000000000028159, PMID: 34941068 PMC8702131

[ref19] Kacaroglu Vicdan A. Gulseven Karabacak B. (2016). Effect of treatment education based on the Roy adaptation model on adjustment of hemodialysis patients. Clinical Nurse Specialist CNS 30, E1–E13. doi: 10.1097/NUR.0000000000000215 27309790

[ref20] Kalfoss M. Schick-Makaroff K. Molzahn A. E. (2019). Living with chronic kidney disease: illness perceptions, symptoms, coping, and quality of life. Nephrol. Nurs. J. 46, 277–290. 31199095

[ref21] Kamran F. Ogden J. (2016). Transitions in psychological well-being and life orientation: the phenomenon of post traumatic growth after renal transplantation. Pak. J. Psychol. Res. 31, 419–440.

[ref22] Knoll G. A. (2013). Kidney transplantation in the older adult. Am. J. Kidney Dis. 61, 790–797. doi: 10.1053/j.ajkd.2012.08.049 23261121

[ref23] Knowles S. R. Castle D. J. Biscan S. M. Salzberg M. O’Flaherty E. B. Langham R. (2016). Relationships between illness perceptions, coping and psychological morbidity in kidney transplants patients. Am J Med Sci 351, 233–238. doi: 10.1016/j.amjms.2015.12.009, PMID: 26992250

[ref24] Langston S. Edwards M. S. Lyvers M. (2018). Illness perceptions, coping, benefit finding, and adjustment in individuals with hepatitis C. Aust. Psychol. 53, 87–96. doi: 10.1111/ap.12255

[ref25] Látos M. Lázár G. Ondrik Z. Szederkényi E. Hódi Z. Horváth Z.. (2022). Positive psychology intervention to improve recovery after renal transplantation: a randomized controlled trial. J. Contemp. Psychother. 52, 35–44. doi: 10.1007/s10879-021-09515-6

[ref26] Li J. Wu X. Lin J. Zou D. Yang X. Cheng S.. (2016). Type D personality, illness perception, social support and quality of life in continuous ambulatory peritoneal dialysis patients. Psychol. Health Med. 22, 196–204. doi: 10.1080/13548506.2016.1224371, PMID: 27550710

[ref27] Lim J. H. Lee G. Y. Jeon Y. Jung H. Y. Choi J. Y. Cho J. H. (2022). Elderly kidney transplant recipients have favorable outcomes but increased infection-related mortality. Kidney Res. Clin. Practice 41, 372–383. doi: 10.23876/j.krcp.21.207 PMC9184840 35286795

[ref28] Livneh H. (2022). Can the concepts of energy and psychological energy enrich our understanding of psychosocial adaptation to traumatic experiences, chronic illnesses and disabilities? Front. Psychol. 13:768664. doi: 10.3389/fpsyg.2022.768664, PMID: 35310232 PMC8927305

[ref29] Livneh H. Antonak R. F. (2005). Psychosocial adaptation to chronic illness and disability: a primer for counselors. J. Couns. Dev. 83, 12–20. doi: 10.1002/j.1556-6678.2005.tb00575.x

[ref30] Liyanage T. Toyama T. Hockham C. Ninomiya T. Perkovic V. Woodward M.. (2022). Prevalence of chronic kidney disease in Asia: a systematic review and analysis. BMJ Glob. Health 7:e007525. doi: 10.1136/bmjgh-2021-007525, PMID: 35078812 PMC8796212

[ref31] Londono Y. McMillan D. E. (2015). Psychosocial adaptation: an evolutionary concept analysis exploring a common multidisciplinary language. J. Adv. Nurs. 71, 2504–2519. doi: 10.1111/jan.12723, PMID: 26148125

[ref32] Lv J. C. Zhang L. X. (2019). Prevalence and disease burden of chronic kidney disease. Adv. Exp. Med. Biol. 1165, 3–15. doi: 10.1007/978-981-13-8871-2_1 31399958

[ref33] Marcos Y. Q. Cantero M. C. T. Escobar C. R. Acosta G. P. (2007). Illness perception in eating disorders and psychosocial adaptation. Eur. Eat. Disord. Rev. 15, 373–384. doi: 10.1002/erv.793, PMID: 17701945

[ref34] Massey E. K. Tielen M. Laging M. Beck D. K. Khemai R. van Gelder T.. (2013). The role of goal cognitions, illness perceptions and treatment beliefs in self-reported adherence after kidney transplantation: a cohort study. J. Psychosom. Res. 75, 229–234. doi: 10.1016/j.jpsychores.2013.07.006, PMID: 23972411

[ref35] Megawati Y. Yetti K. Sukmarini L. (2019). The factors affecting the quality of life of kidney transplantation patients at the Cipto Mangunkusumo general Hospital in Jakarta, Indonesia. Enferm. Clin. 29, 428–433. doi: 10.1016/j.enfcli.2019.04.063

[ref36] Molnar-Varga M. Molnar M. Z. Szeifert L. Kovacs A. Z. Kelemen A. Becze A.. (2011). Health-related quality of life and clinical outcomes in kidney transplant recipients. Am. J. Kidney Dis. 58, 444–452. doi: 10.1053/j.ajkd.2011.03.028 21658828

[ref37] Mouelhi Y. Jouve E. Alessandrini M. Pedinielli N. Moal V. Meurette A.. (2018). Factors associated with health-related quality of life in kidney transplant recipients in France. BMC Nephrol. 19:99. doi: 10.1186/s12882-018-0893-6, PMID: 29703170 PMC5921567

[ref38] Pasquale C. D. (2014). Psychopathological aspects of kidney transplantation: efficacy of a multidisciplinary team. World J. Transplant. 4, 267–275. doi: 10.5500/wjt.v4.i4.267, PMID: 25540735 PMC4274596

[ref39] Pasquale C. D. Pistorio M. L. Veroux M. Indelicato L. Biffa G. Bennardi N.. (2020). Psychological and psychopathological aspects of kidney transplantation: a systematic review. Front. Psych. 11:106. doi: 10.3389/fpsyt.2020.00106, PMID: 32194453 PMC7066324

[ref40] Pisanti R. Poli L. Lombardo C. Bennardi L. Giordanengo L. Berloco P. B.. (2014). The role of transplant-related stressors and social support in the development of anxiety among renal transplant recipients: the direct and buffering effects. Psychol. Health Med. 19, 650–655. doi: 10.1080/13548506.2014.882514, PMID: 24479466

[ref41] Pomey M.-P. Gallego F. B. Affdal A. Fortin M.-C. (2021). Peer mentoring as an avenue to explore in kidney transplantation: kidney transplant recipients’ perspectives on peer mentoring. Transplant. Direct 7:e672:e672. doi: 10.1097/txd.0000000000001130, PMID: 34104710 PMC8183856

[ref42] Pravisani R. Isola M. Baccarani U. Crestale S. Tulissi P. Vallone C. (2021). Impact of kidney transplant morbidity on elderly recipients' outcomes. Aging Clin. Exp. Res. 33, 625–633. doi: 10.1007/s40520-020-01558-4 32323169

[ref43] Sawyer A. T. Harris S. L. Koenig H. G. (2019). Illness perception and high readmission health outcomes. Health Psycho. Open 6:2055102919844504:205510291984450. doi: 10.1177/2055102919844504, PMID: 31041109 PMC6482662

[ref44] Segal O. Goldzweig G. Tako E. Barzilai A. Lyakhovitsky A. Baum S. (2021). Illness perception, perceived social support and quality of life in patients with pemphigus vulgaris: what should dermatologists know? Acta Derm. Venereol. 101:adv00441:adv00441. doi: 10.2340/00015555-3785, PMID: 33723618 PMC9364258

[ref45] Sharma A. Bhardwaj A. Mathur R. P. (2021). Incidence and causes of late hospital readmissions after living donor renal transplant: a retrospective study. Exp. Clin. Transplant. 19, 420–424. doi: 10.6002/ect.2020.0490, PMID: 33877037

[ref46] Singh P. Ng Y. H. Unruh M. (2016). Kidney transplantation among the elderly: challenges and opportunities to improve outcomes. Adv. Chronic Kidney Dis. 23, 44–50. doi: 10.1053/j.ackd.2015.11.002, PMID: 26709062

[ref47] Sitjar-Suñer M. Suñer-Soler R. Masià-Plana A. Chirveches-Pérez E. Bertran-Noguer C. Fuentes-Pumarola C. (2020). Quality of life and social support of people on peritoneal dialysis: mixed methods research. Int. J. Environ. Res. Public Health 17:4240. doi: 10.3390/ijerph17124240, PMID: 32545857 PMC7345330

[ref48] Stoumpos S. Jardine A. G. Mark P. B. (2015). Cardiovascular morbidity and mortality after kidney transplantation. Transpl. Int. 28, 10–21. doi: 10.1111/tri.12413 25081992

[ref49] Suarilah I. Lin C.-C. (2021). Factors influencing self-management among Indonesian patients with early-stage chronic kidney disease: a cross-sectional study. J. Clin. Nurs. 31, 703–715. doi: 10.1111/jocn.15930, PMID: 34405484

[ref50] Thuret R. Timsit M. O. Kleinclauss F. (2016). Insuffisance rénale chronique et transplantation rénale. Prog. Urol. 26, 882–908. doi: 10.1016/j.purol.2016.09.051 27727091

[ref51] Tian X. Gao Q. Li G. Zou G. Liu C. Kong L.. (2016). Resilience is associated with low psychological distress in renal transplant recipients. Gen. Hosp. Psychiatry 39, 86–90. doi: 10.1016/j.genhosppsych.2015.12.004, PMID: 26805002

[ref52] Tzvetanov I. D’Amico G. Walczak D. Jeon H. Garcia-Roca R. Oberholzer J.. (2014). High rate of unemployment after kidney transplantation: analysis of the united network for organ sharing database. Transplant. Proc. 46, 1290–1294. doi: 10.1016/j.transproceed.2014.02.006, PMID: 24836836

[ref53] Vicdan A. K. Karabacak B. G. (2016). Effect of treatment education based on the Roy adaptation model on adjustment of hemodialysis patients. Clin. Nurse Spec. 30, E1–E13. doi: 10.1097/nur.0000000000000215, PMID: 27309790

[ref54] Wang C. Huang Y. Xiao Y. (2021). The mediating effect of social problem-solving between perfectionism and subjective well-being. Front. Psychol. 12:764976. doi: 10.3389/fpsyg.2021.764976, PMID: 34955985 PMC8702494

[ref55] Wang Y. Veltkamp D. M. J. van der Boog P. J. M. Hemmelder M. H. Dekker F. W. de Vries A. P. J.. (2022). Illness perceptions and medication nonadherence to Immunosuppressants after successful kidney transplantation: a cross-sectional study. Transpl. Int. 36:10073. doi: 10.3389/ti.2022.10073, PMID: 35185376 PMC8842226

[ref56] Wurm F. McKeaveney C. Corr M. Wilson A. Noble H. (2022). The psychosocial needs of adolescent and young adult kidney transplant recipients, and associated interventions: a scoping review. BMC Psychol. 10:186:186. doi: 10.1186/s40359-022-00893-7, PMID: 35906706 PMC9336106

[ref57] Xiao P. Liu M. Cui L. Ding S. Xie J. Cheng A. S. (2021). Sexual dysfunction and activity avoidance in female kidney transplant patients. Clin. Transpl. 35:e14363. doi: 10.1111/ctr.14363, PMID: 33998698

[ref58] Xu Y. Liu T. Jiang Y. Zhao X. Meng F. Xu G.. (2022). Psychosocial adaptation among inflammatory bowel disease patients and associated factors: a cross-sectional study. Psychol. Res. Behav. Manag. 15, 2157–2167. doi: 10.2147/prbm.s376254, PMID: 35979227 PMC9377396

[ref59] Yao J. (2013). A Cross-sectional Study on Adaptation of Cancer Patients and Analysis of Its Predictive Factors. Available at: https://kns.cnki.net/kcms/detail/detail.aspx?dbcode=CMFD&dbname=CMFD201401&filename=1013232664.nh&uniplatform=NZKPT&v=UkSYRpeXNCEhemrAabtD9UhfBQhmMXDV860onELVFkmoH67qz6F8x9sV1i-YLp9M ().

[ref60] Yuan J. (2018). Intervention Study of Cognitive Behavior Group on Social Adaptive Ability of Renal Transplantation Patients. master’s thesis. Nanjing (CN): Nanjing University of Science and Technology

[ref61] Zelikovsky N. Nelson E. (2021). Illness perceptions and beliefs about medication: impact on health-related quality of life in adolescent kidney transplant recipients. Pediatr. Transplant. 25:e13988. doi: 10.1111/petr.13988, PMID: 33590948

[ref62] Zelle D. M. Dorland H. F. Rosmalen J. G. M. Corpeleijn E. Gans R. O. B. Homan van der Heide J. J.. (2012). Impact of depression on long-term outcome after renal transplantation: a prospective cohort study. Transplantation 94, 1033–1040. doi: 10.1097/TP.0b013e31826bc3c8, PMID: 23064656

[ref63] Zhang Y. Xian H. Yang Y. Zhang X. Wang X. (2019). Relationship between psychosocial adaptation and health-related quality of life of patients with stoma: a descriptive, cross-sectional study. J. Clin. Nurs. 28, 2880–2888. doi: 10.1111/jocn.14876, PMID: 30939212

[ref64] Zimet G. D. Powell S. S. Farley G. K. Werkman S. Berkoff K. A. (1990). Psychometric characteristics of the multidimensional scale of perceived social support. J. Pers. Assess. 55, 610–617. doi: 10.1080/00223891.1990.9674095 2280326

